# Relationsheep between anxiolytic dependence, anxiety and depression among students from Gabes Institute of Nursing Sciences

**DOI:** 10.1192/j.eurpsy.2025.959

**Published:** 2025-08-26

**Authors:** S. Kolsi, S. Nouili, W. Abbes, W. Ben Flah, A. Bouaziz, K. Medhaffar, W. Abbes

**Affiliations:** 1 Psychiatry, University Hospital, Gabes, Tunisia

## Abstract

**Introduction:**

Access to university marks a crucial stage in the lives of young adults. This transition can generate high levels of stress and anxiety, influencing their mental and emotional well-being.

To overcome these challenges, some students may resort to the increasingly common use of psychotropic drugs, particularly anxiolytics, to ease their distress.

**Objectives:**

To determine the relationship between anxiolytic dependence, anxiety and depression in students.

**Methods:**

This was a cross-sectional, descriptive and analytical study conducted on Gabes institute of nursing sciences, for a period of two months(March to May 2024).

We used:

- Benzodiazepine Attachment Cognitive Scale (BACS) : to study BZD dependence.
  A score above 6 indicates dependance to BZD.
- Hospital and anxiety depression scale (HAD): to assess the severity of anxiety and depressive symptoms. Seven questions relate to anxiety (total A) and seven to depression (total D), giving two scores (maximum score for each = 21).

**Results:**

The sample comprised 33 students. The mean age of our population was 21.30 ±1.51 years and the sex ratio (M/F) was 0.65.

The mean score of the ECAB scale was 6.93, with extremes of [3-10].

According to our results, dependence on BZDs was clearly predominant, found in 28 students (84.8%).

According to the HAD scale, 82% of students showed anxiety symptoms and 24.2% were depressed.

According to our study, we found that BZD dependence in students was strongly correlated with anxiety with p=0.007.

Similarly, we found that those dependent on BZDs were more likely to develop depressive symptoms compared with non-dependents, with a significant difference (p=0.04).

**Conclusions:**

Our findings suggest that the greater the dependence on anxiolytics, the greater the risk of developing anxiety and depressive symptoms.

**Disclosure of Interest:**

None Declared

